# Supplementary material for: Integrating Sequence- and Structure-Based Similarity Metrics for the Demarcation of Multiple Viral Taxonomic Levels
Source: Viruses. 2025 Apr 29;17(5):642. doi: 10.3390/v17050642 (PMC12115509; doi:10.3390/v17050642)
Supplement: Supplementary file 1 [file viruses-17-00642-s001.zip › viruses-3595197-supplementary.pdf]

SUPPLEMENTARY MATERIAL

Integrating sequence- and structure-based similarity metrics for the demarcation of multiple viral taxonomic levels

Igor C. dos Santos [1], Rebecca di Stephano de Souza[2], Igor Tolstoy[3], Liliane S. Oliveira[4] and Arthur Gruber [5,6*]

[1] Biotechnology undergraduate course, Escola de Artes, Ciências e Humanidades, Universidade de São Paulo, São Paulo, Brazil; igor223tgec@gmail.com
[2] Biological Sciences undergraduate course, Instituto de Biociências, Universidade de São Paulo, São Paulo, Brazil; rebeccadistephano@usp.br
[3] Argentys Informatics, LLC, 12 South Summit Avenue Suite 200, Gaithersburg, MD 20877, USA; itolstoy@gmail.com
[4] Department of Computer Science, Federal University of Technology of Paraná (UTFPR), Alberto Carazzai Avenue, 1640, Cornélio Procópio 86300-000, PR, Brazil
[5] Department of Parasitology, Instituto de Ciências Biomédicas, Universidade de São Paulo, São Paulo, SP, 05508-000, Brazil; argruber@usp.br
[6] Affiliated member of the European Virus Bioinformatics Center, Leutragraben 1, Jena, 07743, Germany
* Correspondence: argruber@usp.br (AG); Tel.: 55 11 3091-7274

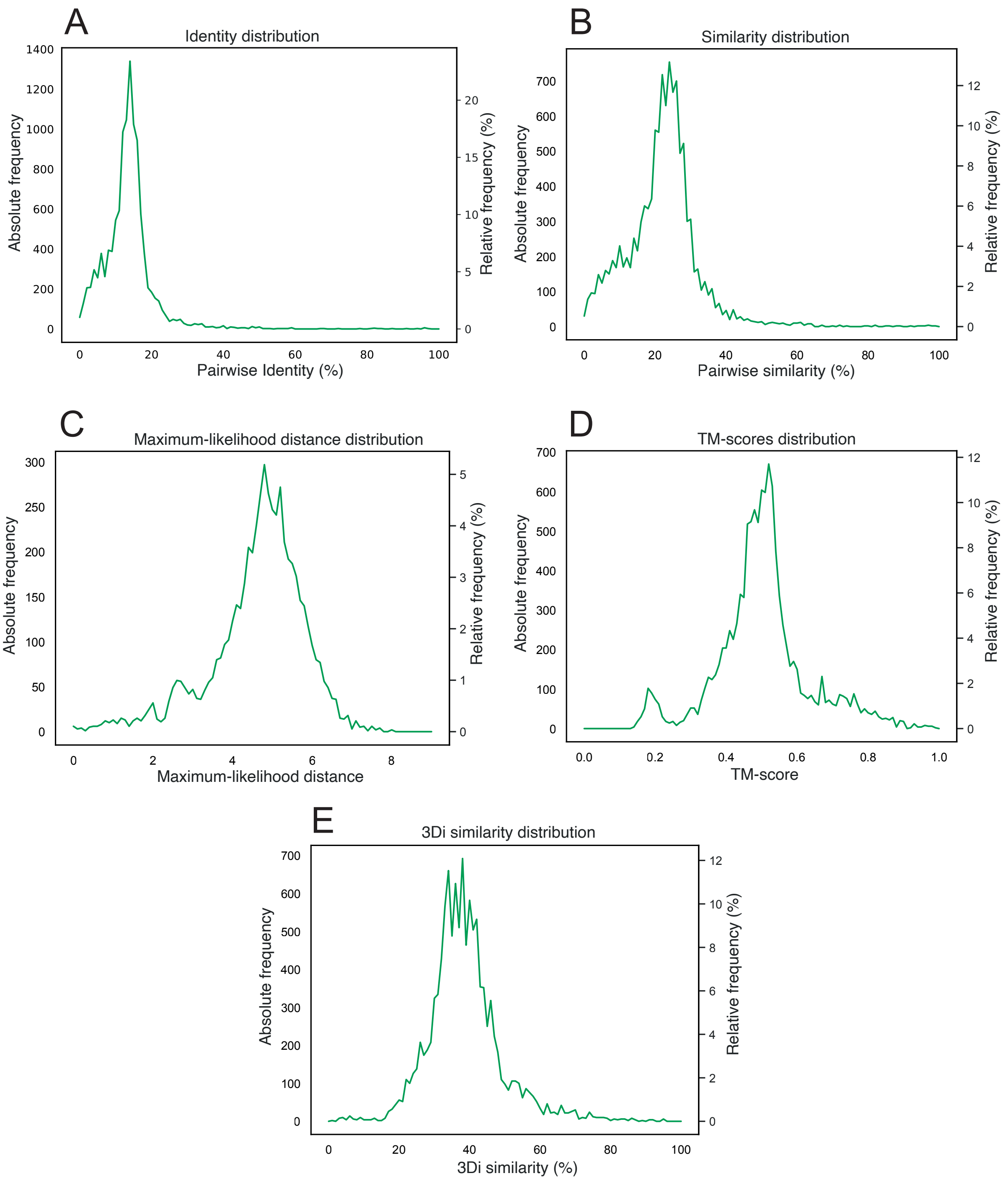

Figure S1 – Frequency distribution plots of all-against-all pairwise comparisons of RDRP amino acid sequences of RNA virus families of the *Orthornavirae* kingdom. The plots are derived from different metrics: identity (A) and similarity (B) percentages and maximum likelihood distance (C) of amino acid sequences, TM-scores of 3D structures (D), and 3Di-character sequence similarity (E).

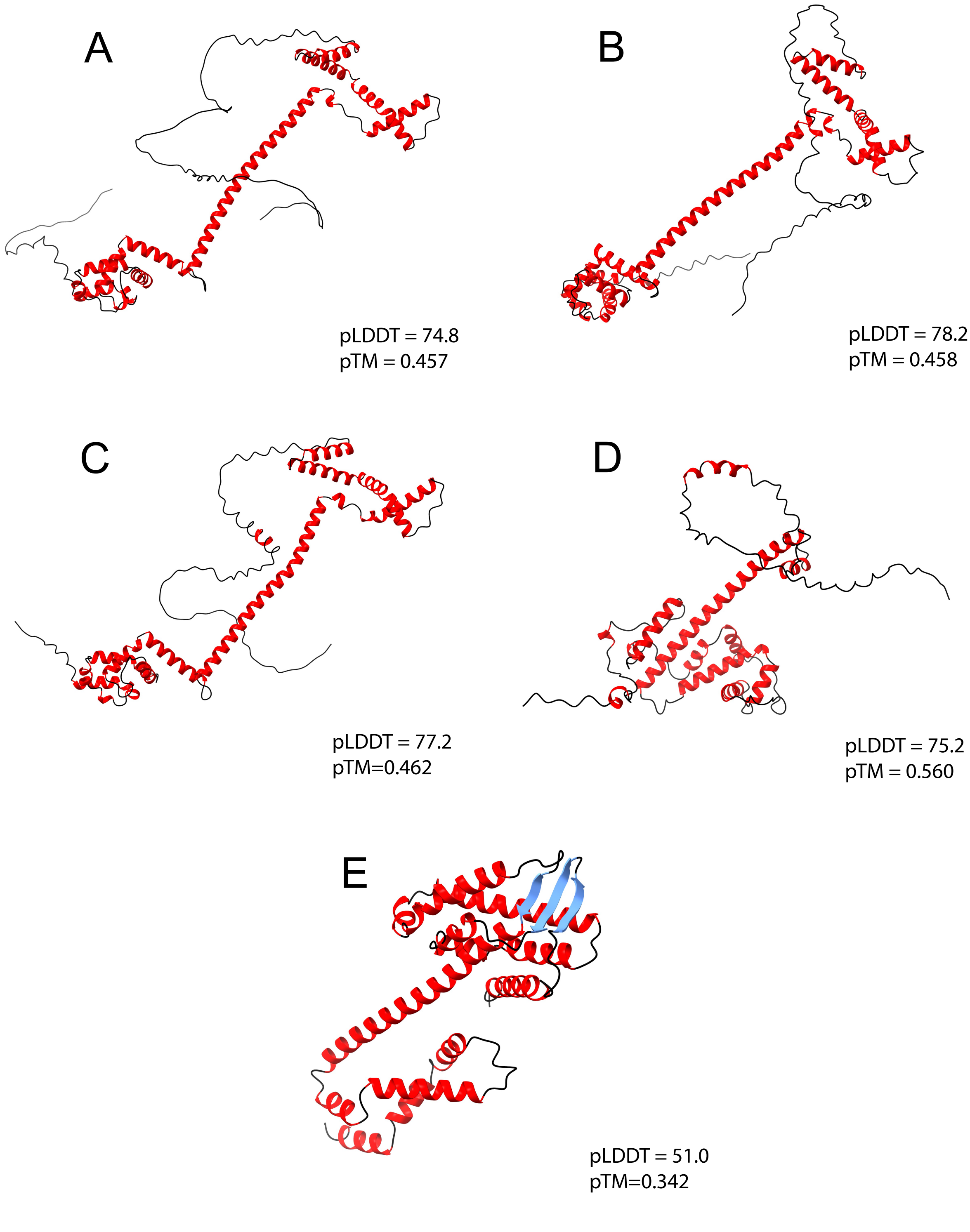

Figure S2. Three-dimensional structures of the RDRP of representative viruses of the *Amalgaviridae* family: (A) Rhododendron virus A - YP_003868437; (B) Southern Tomato Virus - AVG19367; (C) Blueberry latent virus - ADO14117; (D) Penicillium aurantiogriseum bipartite virus 1 - ALO501291; (E) Zygosaccharomyces bailii virus Z - NP_624324. The 3D structures were predicted with AlphaFold2 using the ColabFold v1.5.5 server: AlphaFold2.

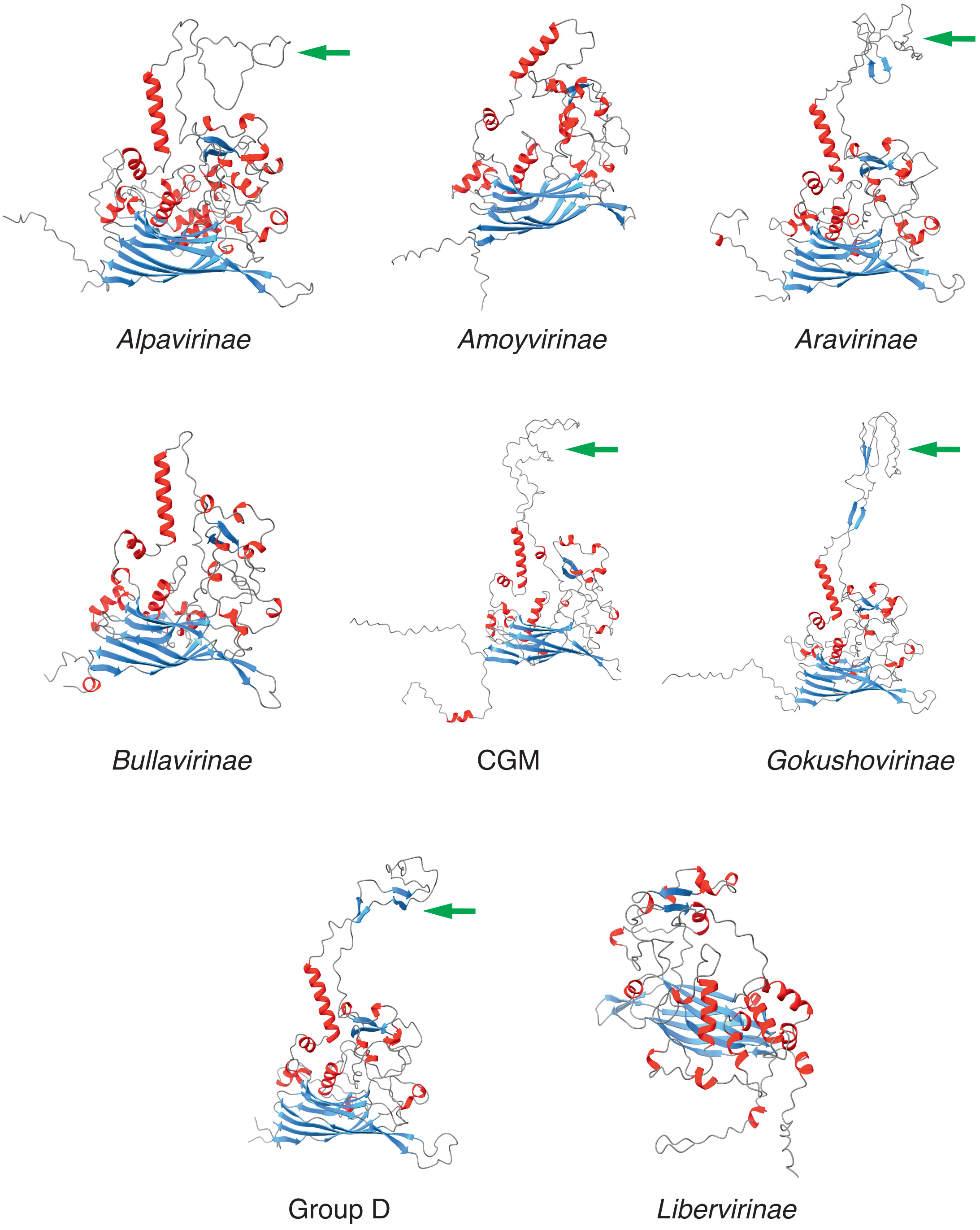

Figure S3. Three-dimensional structures of VP1 (major capsid protein) of the subfamilies/groups of the *Microviridae* family. The 3D structures were predicted with AlphaFold2 using the ColabFold v1.5.5 server: AlphaFold2. The green arrows indicate protrusion domains that constitute mushroom-like structures in the viral capsid.

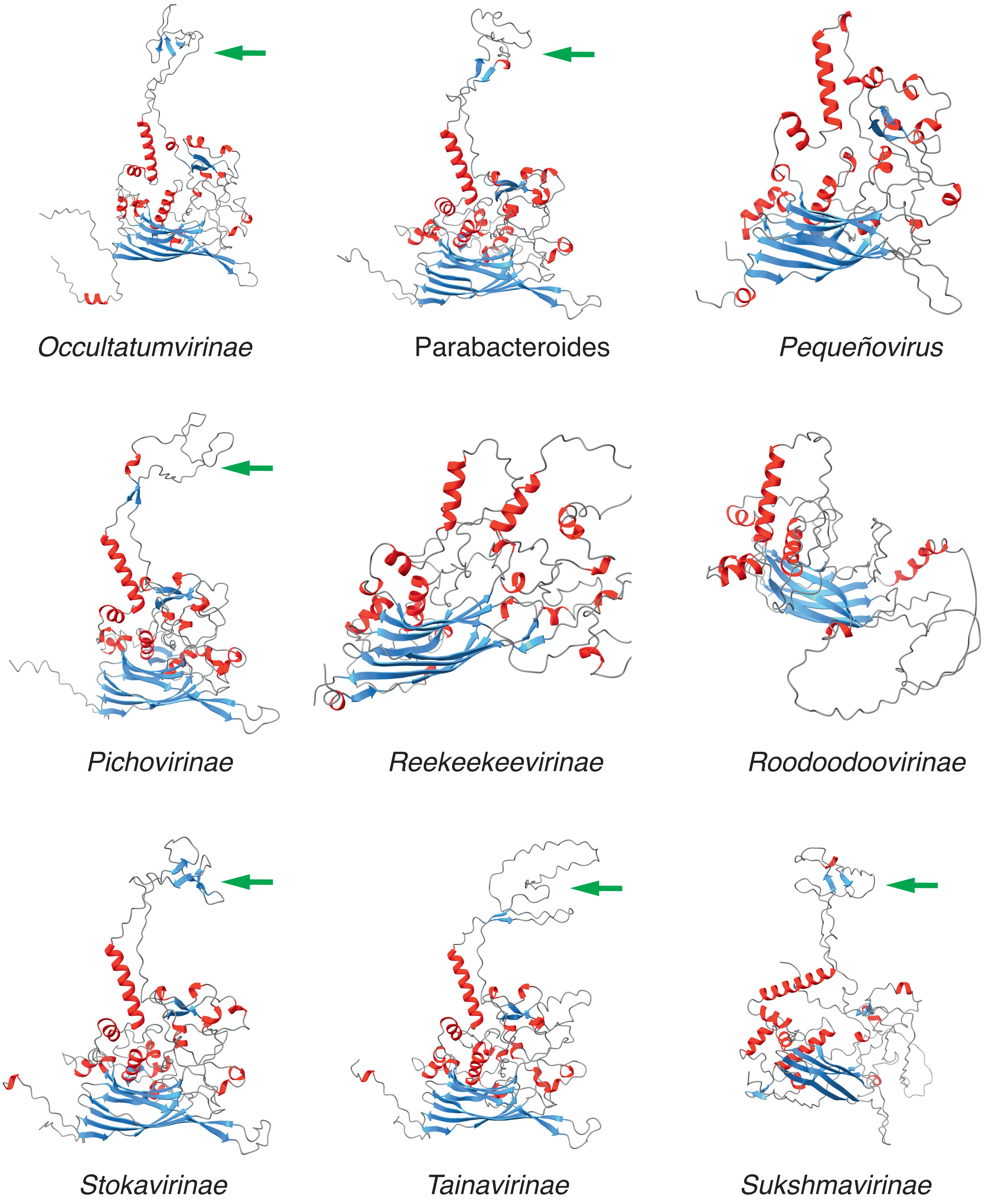

Figure S3 - continuation. Three-dimensional structures of VP1 (major capsid protein) of the subfamilies/groups of the *Microviridae* family. The 3D structures were predicted with AlphaFold2 using the ColabFold v1.5.5 server: AlphaFold2. The green arrows indicate protrusion domains that constitute mushroom-like structures in the viral capsid.

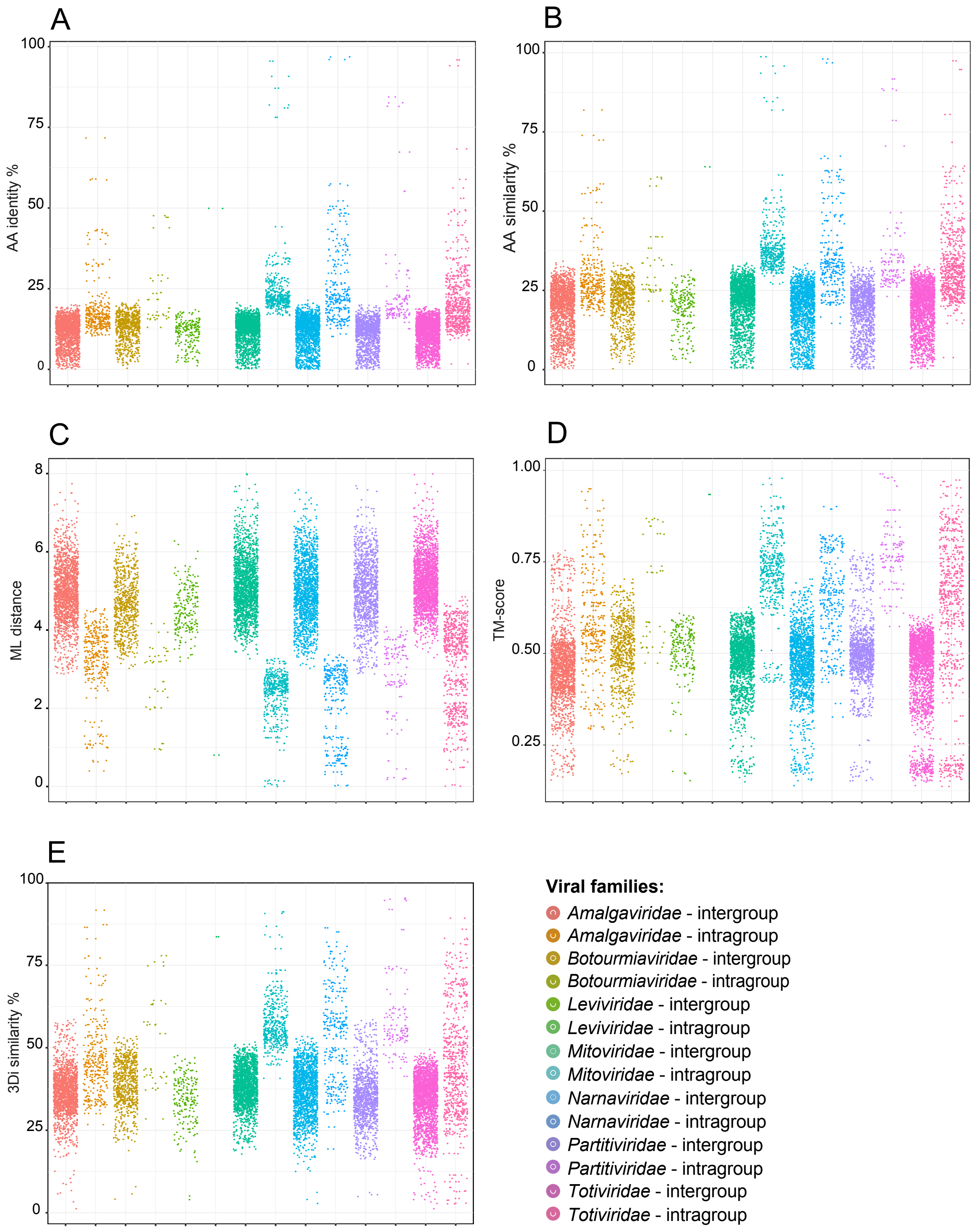

Figure S4. All-against-all pairwise comparisons of RDRP amino acid sequences of viral families of the *Orthornavirae* kingdom. Members of each viral family were defined by a maximum likelihood phylogenetic reconstruction using MAFFT and IQ-TREE 2, resulting in *bona fide* subsets. For each subset, sequences were compared all-against-all using identity (A) and similarity (B) percentages and maximum likelihood distance (C) of amino acid sequences, TM-scores of 3D structures (D), and 3Di-character sequence similarity (E). The dots of each column of the scatter plot depict the results obtained for intergroup and intragroup pairwise comparisons, respectively.

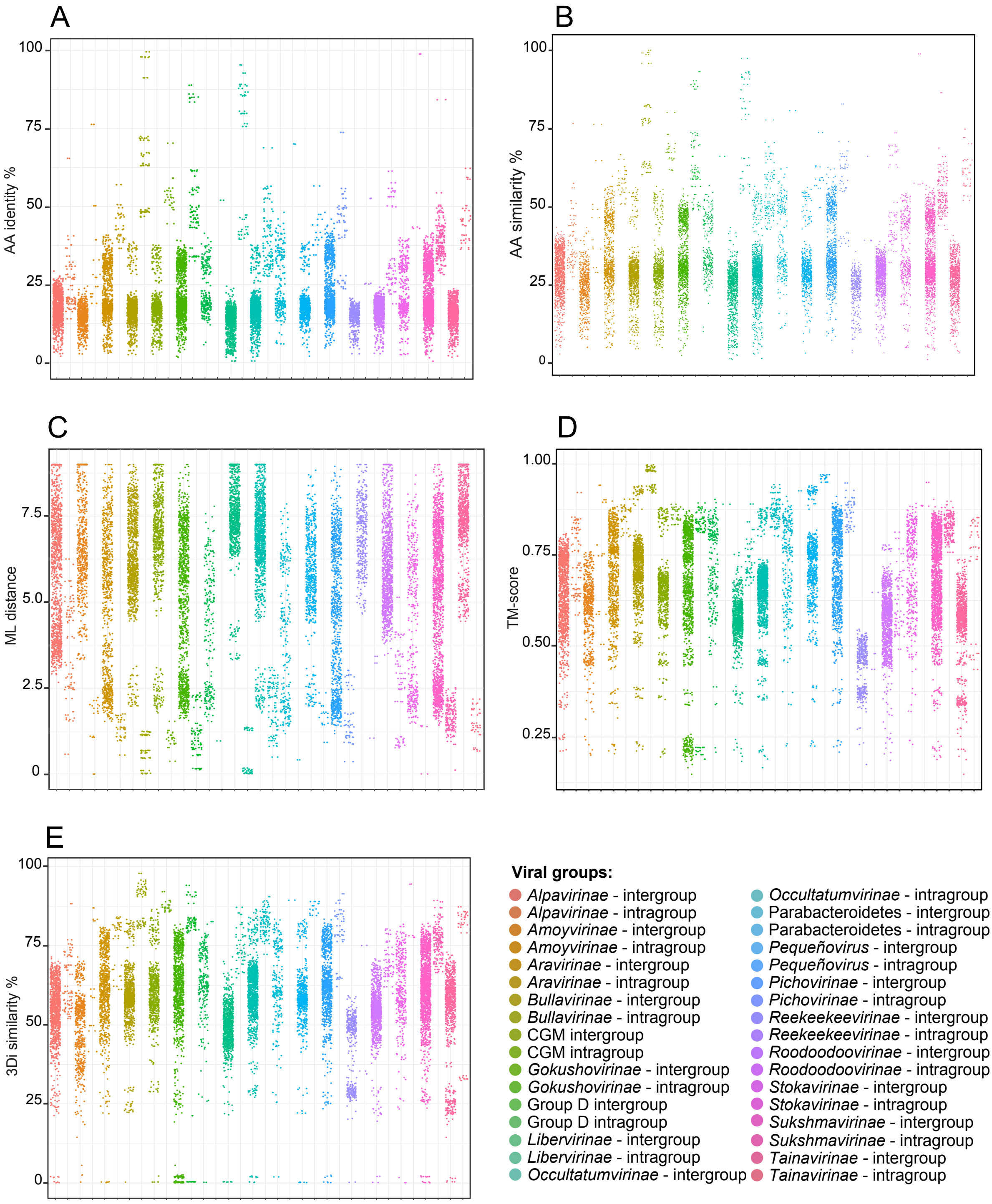

Figure S5. All-against-all pairwise comparisons of VP1 amino acid sequences of *Microviridae* phages. Members of each viral group were defined by a maximum likelihood phylogenetic reconstruction using MAFFT and IQ-TREE 2, resulting in *bona fide* subsets. For each subset, sequences were compared all-against-all using identity (A) and similarity (B) percentages and maximum likelihood distance (C) of amino acid sequences, TM-scores of 3D structures (D), and 3Di-character sequence similarity (E). The dots of each column of the scatter plot depict the results obtained for intergroup and intragroup pairwise comparisons, respectively.

.

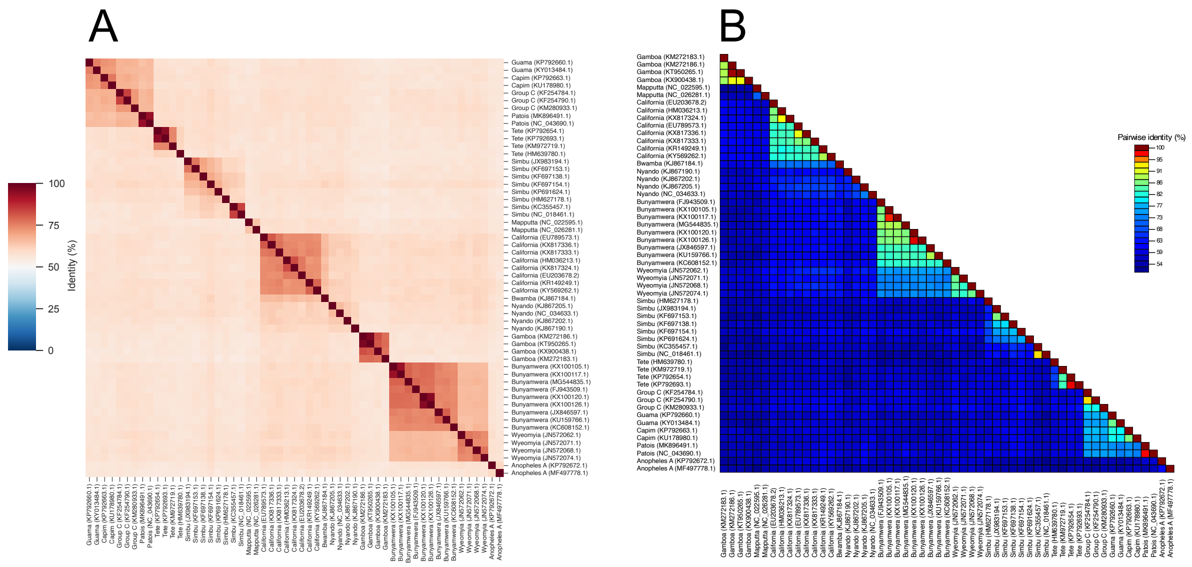

Figure S6 - Heatmaps generated by MPACT (A) and SDT (B) programs of all-against-all pairwise comparisons of the large (L) segment of viruses of the genus *Orthobunyavirus* using identity percentage of nucleotide sequences.

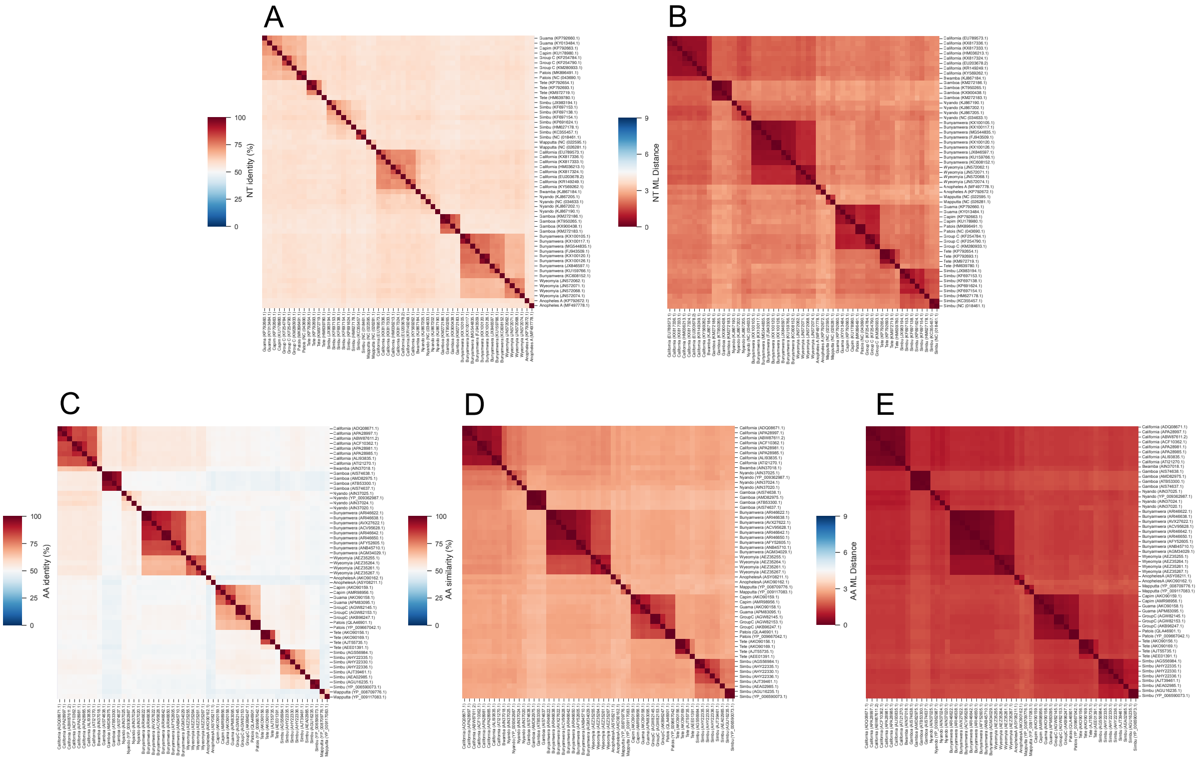

Figure S7. Heatmaps of all-against-all pairwise comparisons of sequences derived from the large (L) segment of viruses of the genus *Orthobunyavirus*. Sequences were compared all-against-all using pairwise identity percentage (A) and maximum likelihood distance (B) of nucleotide sequences, pairwise identity (C) and similarity (D) percentages, and maximum likelihood distance (E) of amino acid sequences.

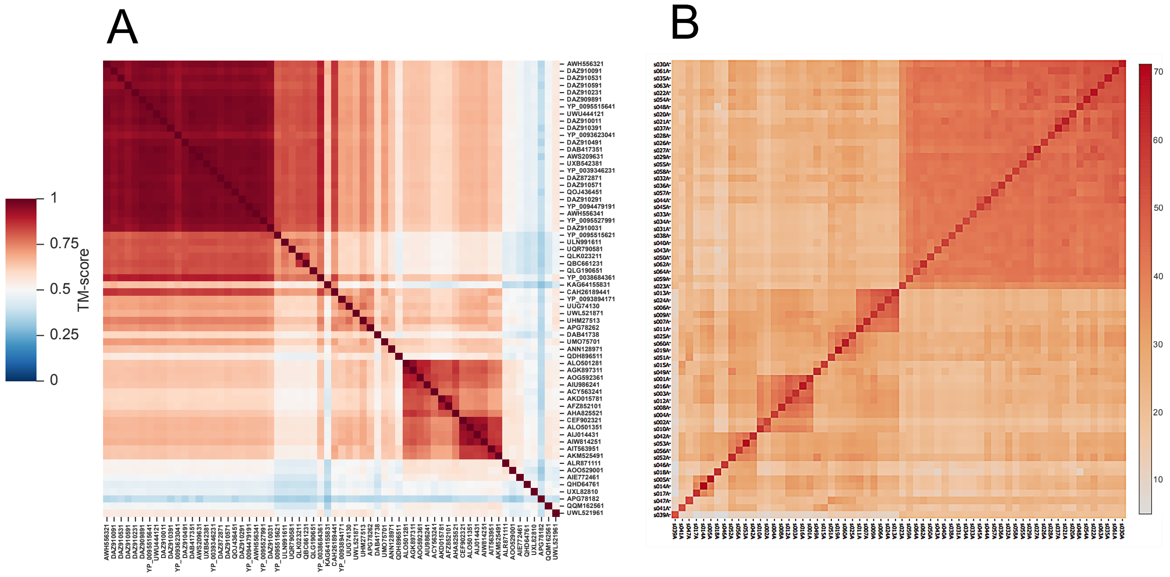

Figure S8 - Comparison of heatmaps generated by MPACT (A) and Dali (B). Heatmaps were generated using a dataset of 64 PDB structures predicted by AlphaFold 2 using RDRP sequences of viruses of the *Amalgaviridae* family. Heatmaps are derived from pairwise structural alignments using TM-scores obtained with TM-align (A) and structural similarity Z-scores using Dali (B).

Table S1. Amino acid sequences of the RDRP of viruses of the *Orthornavirae* kingdom

Organism	Accession code*
Aedes japonicus narnavirus 1	QIP67846
Alternaria arborescens mitovirus 1	YP_009270635
Antonospora locustae virus 1	YP_009389417
Aspergillus fumigatus mitovirus 1	AXE72932
Aspergillus ochraceous virus	ABV30675
Atkinsonella hypoxylon virus	AAA61829
Barns Ness breadcrumb sponge narna-like virus 4	ASM94069
Beauveria bassiana RNA virus 1	CEF90232
Beauveria bassiana victorivirus 1	YP_009508251
Beihai narna-like virus 18	YP_009333315
Beihai narna-like virus 9	YP_009333316
Binucleate Rhizoctonia mitovirus K1	YP_009165597
Black raspberry virus F	YP_001497151
Blechomonas maslovi Leishmaniavirus-like RNA virus 1	AYD61662
Blueberry latent virus	YP_003934623
Buergenerula spartinae mitovirus 1	AHY03257
Carrot cryptic virus	ACL93278
Cassava virus C	YP_003104770
Ceratocystis polonica partitivirus	YP_001911122
Colletotrichum higginsianum RNA virus 1	AIW81425
Coniothyrium minitans RNA virus	WAK77537
Coquillettidia venezuelensis narnavirus 2	QBA55487
Cryphonectria parasitica mitovirus 1-NB631	NP_660174
Cryptosporidium parvum virus 1	AAC47805
Culex narnavirus 1	QBR53296
Drosophila melanogaster totivirus SW-2009a	YP_003289293
Eimeria brunetti RNA virus 1	NP_108651
Eimeria stiedai GUNCC_DN54384-11 RNA virus 1	QIJ70039
Eimeria stiedai TQCBD-12/0909 RNA virus 1	YP_009551684
Eimeria tenella RNA virus 1	YP_009115500
Epirus cherry virus	YP_002019754
Escherichia phage MS2	YP_009640127
Escherichia virus BZ13	NP_040755
Exobasidium gracile zybavirus 1-3	UWL52196
Fusarium graminearum dsRNA mycovirus 4	ACY56324
Fusarium poae mitovirus 4	YP_009272901
Giardia canis virus	ABB36743
Giardia lamblia virus	NP_620070
Grapevine associated narnavirus-1	CEZ26304

Grapevine associated narnavirus-1	YP_009182162
Gremmeniella abietina RNA virus 6	AIU98624
Gremmeniella abietina RNA virus L1	NP_624332
Gremmeniella abietina RNA virus MS1	NP_659027
Gutsystermes virus	QQM16351
Haverford narna-like virus	QIJ70061
Hubei narna-like virus 3	YP_009337787
Lutzomyia longipalpis mitovirus 1	DAD49837
MAG Totiviridae	UHS72566
MAG Totiviridae	UHS72498
Magnaporthe oryzae virus 1	YP_122352
Mitovirus AEF-2013	AGW51760
Narnaviridae environmental sample	AJT39596
Nigrospora oryzae RNA virus 1	ALR87111
Ochlerotatus-associated narna-like virus 1	AGW51766
Ochlerotatus-associated narna-like virus 2	AGW51768
Omono River virus	BAJ21511
Ophiostoma mitovirus 4	NP_660179
Ourmia melon virus	YP_002019757
Penaeid shrimp infectious myonecrosis virus	AAT67231
Penicillium aurantiogriseum virus 1	ALO50128
Penicillium janczewskii virus	ALO50135
Penicillium stoloniferum virus F	YP_271922
Pepper cryptic virus 1	AEJ07890
Pichia membranifaciens virus L-A	UNY77798
Plasmopara viticola mitovirus 23	QIR30246
Plasmopara viticola mitovirus 39	QIR30262
Plasmopara viticola mitovirus 56	QIR30279
Plasmopara viticola narnavirus 17	QIR30296
Psorophora varipes narnavirus	QBA55486
Purpureocillium lilacinum virus 1	AOO52900
Radish partitivirus JC-2004	AAU88207
Rhizoctonia fumigata mycovirus	AKD01578
Rhizoctonia solani mitovirus 28	QDW65418
Rhizoctonia solani mitovirus 30	QDW65420
Rhizoctonia solani virus 717	NP_620659
Rhizopus microsporus 20S narnavirus	QBC65280
Rhizopus microsporus 23S narnavirus	QBC65281
Rhododendron virus A	YP_003868436
Saccharomyces 20S RNA narnavirus	NP_660178
Saccharomyces 23S RNA narnavirus	AAC98708
Saccharomyces cerevisiae virus L-A	NP_620495
Scheffersomyces segobiensis virus L	YP_009507829
Sclerotinia sclerotiorum mitovirus 1 HC025	YP_009121785

Sclerotinia sclerotiorum mitovirus 1 HC025-A	AWY10963
Sclerotinia sclerotiorum mitovirus 1-A2	AWY10962
Setosphaeria turcica mitovirus 1	AZT88625
Southern tomato virus	QLG19065
Soybean leaf-associated mitovirus 2	ALM62242
Sphaeropsis sapinea RNA virus 1	NP_047558
Tianjin totivirus	AFE02920
Totiviridae	QXV86392
Trichomonas vaginalis virus 2	AET81016
Trichomonas vaginalis virus 3	AAL37370
Trichomonas vaginalis virus 4	AED99796
Tuber excavatum mitovirus	AEP83726
Tynnyfer narna-like virus	QIJ70064
Ustilaginoidea virens RNA virus M	AIT56395
Ustilaginoidea virens virus 1	AIE77246
Vicia cryptic virus	YP_272124
Vicia cryptic virus M	QLK02321
Wenling narna-like virus 9	YP_009337200
White clover cryptic virus 1	AAU14888
Wuhan insect virus 18	YP_009342440
Xanthophyllomyces dendrorhous virus L1A	YP_007697651
Zhejiang mosquito virus 3	YP_009333331
Zhejiang mosquito virus 3	ASA47481
Zygosaccharomyces bailii virus Z	ANN12897

* NCBI Protein - https://www.ncbi.nlm.nih.gov/protein/

Table S2. ORF 1 protein sequences of *Amalgaviridae* viruses

Organism	Accession code
Acer pseudosieboldianum amalgavirus 1	DAZ90985.1
Allium cepa amalgavirus 1	YP_009447919.1
Alternaria longipes dsRNA virus 1	AIJ01442.1
Amaranthus tuberculatus amalgavirus 1	DAZ90989.1
Amaranthus tuberculatus amalgavirus 2	DAZ90991.1
Amaranthus tuberculatus amalgavirus 3	DAZ90993.1
Antonospora locustae virus 1	YP_009389418.1
Arceuthobium sichuanense virus 4	DAZ87287.1
Beauveria bassiana RNA virus 1	CEF90231.1
Beihai barnacle virus 14	APG78181.1
Blueberry latent virus	ADO14117.1
Camellia oleifera amalgavirus 1	YP_009551564.1
Cannabis sativa amalgavirus 1	DAZ91001.1
Capillidibolus rugosus virus 1	UXL82811.1
Cistus incanus RNA virus 1	AWS20963.1
Cleome droserifolia amalgavirus 1	YP_009553342.1
Colletotrichum higginsianum dsRNA virus 1	AIW81424.1
Cryphonectria parasitica bipartite mycovirus 1	AGK89732.1
Curvularia thermal tolerance virus	ABM92660.1
Epimedium pseudowushanense amalgavirus 1	DAZ91003.1
Epipogium amalgavirus 1	UWU44412.1
Erigeron breviscapus amalgavirus 1	YP_009552087.1
Exobasidium gracile zybavirus 1-3	UWL52196.1
Exobasidium gracile zybavirus 2-3	UWL52187.1
Ferula assa-foetida amalgavirus 1	DAZ91007.1
Festuca pratensis amalgavirus 1	YP_009552799.1
Fusarium graminearum dsRNA mycovirus 4	ACY56325.1
Guizotia abyssinica amalgavirus 1	DAZ91009.1
Gymnadenia amalgavirus 1	UXB54238.1
Hubei partiti-like virus 59	KX884149.1
Itsystermes virus	QQM16255.1
Lolium perenne amalgavirus 1	DAB41735.1
Medicago sativa amalgavirus 1	QOJ43645.1
Melastoma malabathricum amalgavirus 1	DAZ91029.1
Melilotus officinalis amalgavirus 1	DAZ91031.1
Myosoton aquaticum amalgavirus 1	DAZ91023.1
Nigrospora oryzae unassigned RNA virus 1	ALR87112.1
Paris polyphylla amalgavirus 1	DAZ91049.1
Penicillium aurantiogriseum bipartite virus 1	ALO50129.1
Penicillium janczewskii Beauveria bassianalike virus 1	ALO50134.1

Pinus albicaulis amalgavirus 1	DAZ91039.1
Pinus patula amalgavirus 1	DAB41737.1
Plasmopara viticola lesion associated amalga like virus 1	QHD64762.1
Purpureocillium lilacinum nonsegmented virus 1	AOO52901.1
Rhizoctonia fumigata mycovirus	AKD01579.1
Rhizoctonia solani dsRNA virus 1	AFZ85211.1
Rhodiola rosea amalgavirus 1	DAZ91053.1
Rhododendron virus A	YP_003868437.1
Rubber dandelion latent virus 1	AWH55634.1
Rubber dandelion latent virus 2	AWH55632.1
Salicornia europaea amalgavirus 1	DAB41669.1
Salvia miltiorrhiza amalgavirus 1	DAZ91057.1
Sanya amalgavirus 1	UHM27512.1
Sclerotium hydrophilum virus 1	AOG59237.1
Secale cereale amalgavirus 1	DAB41729.1
Southern tomato virus	AVG19367.1
Spinach amalgavirus 1	YP_009388304.1
Spinacia oleracea amalgavirus 2	DAZ91059.1
Tetrodontophora bielanensis associated virus 1	GAXI02024802.1
Ustilaginoidea virens nonsegmented virus 1	AIE77247.1
Ustilaginoidea virens unassigned RNA virus HNND1	AKM52548.1
Vicia cryptic virus M	QLK02322.1
XiangYun partiti-picobirna-like virus 7	UUG74129.1
Xisha Islands zybavirus	UMO75700.1
Zostera marina amalgavirus 1	YP_009362302.1
Zygosaccharomyces bailii virus Z	NP_624324.1

Table S3. RNA-directed RNA polymerase sequences of *Amalgaviridae* viruses

Organism	Accession code
Allium cepa amalgavirus 1	YP_009447919.1
Alternaria longipes dsRNA virus 1	AIJ01443.1
Amaranthus tuberculatus amalgavirus 1	DAZ90989.1
Anthoxanthum odoratum amalgavirus 1	YP_009551562.1
Anthoxanthum odoratum amalgavirus 2	DAB41668.1
Antonospora locustae virus 1	YP_00938941
Arceuthobium sichuanense virus 4	DAZ87287.1
Beauveria bassiana RNA virus 1	CEF90232.1
Beihai barnacle virus 14	APG78182.1
Blueberry latent virus	YP_003934623.1
Camellia oleifera amalgavirus 1	YP_009551564.1
Cannabis sativa amalgavirus 1	DAZ91001.1
Capillidibolus rugosus virus 1	UXL82810.1
Cistus incanus RNA virus 1	AWS20963.1
Cleome droserifolia amalgavirus 1	YP_009553342.1
Colletotrichum higginsianum nonsegmented dsRNA virus 1	AIW81425.1
Cryphonectria parasitica bipartite mycovirus 1	AGK89731.1
Cucumis melo amalgavirus 1	QBC66123.1
Epimedium pseudowushanense amalgavirus 1	DAZ91003.1
Epipogium amalgavirus 1	UWU44412.1
Exobasidium gracile zybavirus 1-3	UWL52196.1
Exobasidium gracile zybavirus 2-3	UWL52187.1
Festuca pratensis amalgavirus 1	YP_009552799.1
Fusarium graminearum dsRNA mycovirus 4	ACY56324.1
Gremmeniella abietina RNA virus 6	AIU98624.1
Guizotia abyssinica amalgavirus 1	DAZ91009.1
Gymnadenia amalgavirus 1	UXB54238.1
Heterobasidion RNA virus 6	AHA82552.1
Hubei partiti-like virus 59	APG78262.1
Itsystermes virus	QQM16256.1
Lily amalgavirus 1	UQR79058.1
Lolium perenne amalgavirus 1	DAB41735.1
Lycopod associated amalgavirus	CAH2618944.1
Medicago sativa amalgavirus 1	QOJ43645.1
Melastoma malabathricum amalgavirus 1	DAZ91029.1
Myosoton aquaticum amalgavirus 1	DAZ91023.1
Nigrospora oryzae unassigned RNA virus 1	ALR87111.1
Paris polyphylla amalgavirus 1	DAZ91049.1
Partitiviridae sp.	QDH89651.1
Penicillium aurantiogriseum bipartite virus 1	ALO50128.1

Penicillium janczewskii Beauveria bassianalike virus 1	ALO50135.1
Pinus albicaulis amalgavirus 1	DAZ91039.1
Plasmopara viticola lesion associated amalga like virus 1	QHD64761.1
Pterostylis amalgavirus 1	ULN99161.1
Purpureocillium lilacinum nonsegmented virus 1	AOO52900.1
Rhizoctonia fumigata mycovirus	AKD01578.1
Rhizoctonia solani dsRNA virus 1	AFZ85210.1
Rhodiola rosea amalgavirus 1	DAZ91053.1
Rhododendron virus A	YP_003868436.1
Rubber dandelion latent virus 1	AWH55634.1
Rubber dandelion latent virus 2	AWH55632.1
Salicornia europaea amalgavirus 1	DAB41669.1
Salvia miltiorrhiza amalgavirus 1	DAZ91057.1
Salvia splendens	KAG6415583.1
Sanya amalgavirus 1	UHM27513.1
Sclerotium hydrophilum virus 1	AOG59236.1
Southern tomato virus	QLG19065.1
Spinacia oleracea amalgavirus 2	DAZ91059.1
Tetrodontophora bielanensis associated virus 1	DAB41738.1
Ustilaginoidea virens nonsegmented virus 1	AIE77246.1
Ustilaginoidea virens RNA virus M	AIT56395.1
Ustilaginoidea virens unassigned RNA virus HNND1	AKM52549.1
Vicia cryptic virus M	QLK02321.1
XiangYun partiti-picobirna-like virus 7	UUG74130.1
Xisha Islands zybavirus	UMO75701.1
Zostera marina amalgavirus 2	YP_009362304.1
Zygosaccharomyces bailii virus Z	ANN12897.1

Table S4. Amino acid sequences of the VP1 of *Microviridae* phages

**Organism**	**Accession code**	**Source**
167126con0073	Unavailable	[a]F. Enault
167127con0067	Unavailable	[a]F. Enault
59_Coral_002	Unavailable	[b]Roux *et al.*
7789_ LSPY01000125_gene_2	Unavailable	[c]Metavir
7789_LSPY01000141_gene_2	Unavailable	[c]Metavir
7789_LSPY01000142_gene_4	Unavailable	[c]Metavir
7789_LSPY01000149_gene_4	Unavailable	[c]Metavir
7789_LSPY01000166_gene_5	Unavailable	[c]Metavir
7790_LSPZ01000286_gene_2	Unavailable	[c]Metavir
7790_LSPZ01000292_gene_7	Unavailable	[c]Metavir
7793_LSQA01000331_gene_3	Unavailable	[c]Metavir
Bacteroides thetaiotaomicron strain 19_BTHE	JVQR01000241	[d]BV-BRC
Bacteroides thetaiotaomicron strain MCC174	WQQQ01000002	[d]BV-BRC
Bog1249_12	AKI26871	[e]NCBI
Bog5275_51	AKI26877	[e]NCBI
Bog9017_22	AKI26894	[e]NCBI
Bourget_523	Unavailable	[b]Roux *et al.*
C. tardaugens	WP_021691789	[e]NCBI
C. tardaugens NBRC 16725	CP034179	[e]NCBI
Candidatus L. asiaticus	UDE22491	[e]NCBI
Candidatus L. asiaticus	WP_045490387	[e]NCBI
Candidatus L. solanacearum	WP_013462096	[e]NCBI
Candidatus L. solanacearum	WP_013462103	[e]NCBI
Candidatus L. solanacearum	WP_034441566	[e]NCBI
Candidatus L. solanacearum	WP_045960446	[e]NCBI
Candidatus L. solanacearum	WP_103847521	[e]NCBI
Candidatus L. solanacearum	WP_240535991	[e]NCBI
Candidatus Prevotella intestinigallinarum strain 146	DXID01000046	[d]BV-BRC
Candidatus Rhodobacter oscarellae	WP_049643002	[e]NCBI
CGM_222	AXL15495	[e]NCBI
CGM_223	AXL15500	[e]NCBI
CGM_249	AXL15623	[e]NCBI
CGM_250	AXL15627	[e]NCBI
CGM_251	AXL15631	[e]NCBI
CGM_252	AXL15635	[e]NCBI
Citromicrobium vB_Cib_ssDNA_P1	ATW62973	[e]NCBI
Clostridia bacterium strain RGIG9399	JAGBSF010000052	[d]BV-BRC
Clostridium sp. CAG:306	FR888562	[e]NCBI
Dragonfly-associated microphage 1	YP_006908226	[e]NCBI
Dysgonomonas macrotermitis strain DSM 27370	FQUC01000005	[d]BV-BRC
Eel River basin pequenovirus	AJK28249	[e]NCBI

Eel River basin pequenovirus	AJK28267	[e]NCBI
Eel River basin pequenovirus	AJK28290	[e]NCBI
Eel River basin pequenovirus	AJK28332	[e]NCBI
Eel River basin pequenovirus	YP_009126958	[e]NCBI
Elizabethkingia anophelis NUH6	ASYJ01000020	[d]BV-BRC
Elizabethkingia meningoseptica strain CSID_3000515919	MAGZ01000012	[d]BV-BRC
Enterobacter aerogenes phage strain 33850	LAAO01000012	[d]BV-BRC
Enterobacter aerogenes strain GN05905	LVUT01000012	[d]BV-BRC
Enterobacter ludwigii strain 120152	JAHEUQ010000001	[d]BV-BRC
Enterobacteria phage NC11	AAZ49145	[e]NCBI
Enterobacteria phage WA6	AAZ49332	[e]NCBI
Epibacterium mobile A3R06	JAHKQQ010000030	[d]BV-BRC
Escherichia coli strain 1724	PIJS01000026	[d]BV-BRC
Escherichia coli strain A7	JAFJVT010000082	[d]BV-BRC
Escherichia coli strain SI-NP049	BGHX01000135	[d]BV-BRC
Escherichia phage alpha3	NP_039597.1	[e]NCBI
Escherichia phage G4	YP_009497844.1	[e]NCBI
Escherichia phage phiK	NP_0439491	[e]NCBI
Escherichia phage phiK	NP_043949.1	[e]NCBI
Escherichia phage phiX174	NP_0407111	[e]NCBI
Escherichia phage St-1	YP_002985212.1	[e]NCBI
Fen2266_1	AKI26898	[e]NCBI
Fen418_41	AKI26903	[e]NCBI
Fen4707_41	AKI26908	[e]NCBI
Fen51_42	AKI26914	[e]NCBI
Fen685_11	AKI26926	[e]NCBI
Fen7786_21	AKI26931	[e]NCBI
Fen7895_21	AKI26942	[e]NCBI
Fen7940_21	YP_009160412	[e]NCBI
JCVI_003	Unavailable	[b]Roux *et al.*
Kiritimatiellae bacterium strain MAG40	JADBNW010000027	[d]BV-BRC
Leptospirales bacterium strain Th196P4bin55	WRNR01000003	[d]BV-BRC
MAG	AXF52647	[e]NCBI
MAG	AXH73930	[e]NCBI
MAG	AXH75895	[e]NCBI
MAG	AXH76151	[e]NCBI
MAG	AXH78112	[e]NCBI
MAG Sphingomonas sp. 67-3	OJV31495	[e]NCBI
Maritimibacter alexandrii	WP_138424419	[e]NCBI
Muribaculaceae bacterium MGYG-HGUT-02556	CABMOX010000001	[d]BV-BRC
Oceanicola sp.	WP_010139563	[e]NCBI
Oscillibacter sp. PC13	FOXE01000003	
Parabacteroides phage YZ-2015a	AKI26957	[e]NCBI

Parabacteroides phage YZ-2015b	AKI26963	[e]NCBI
Pavin_279	Unavailable	[b]Roux *et al.*
Pavin_723	Unavailable	[b]Roux *et al.*
Prevotella sp. oral taxon 317 str. F0108	NZ_GG740072	[e]NCBI
Prevotella sp. strain RGIG2735	JAFRRN010000061	[d]BV-BRC
Rhizobium phage RHph_TM1_10A	QIG67604	[e]NCBI
Rhizobium phage RHph_TM24	QIG67103	[e]NCBI
Rhodobacter capsulatus DE442	AYPR01000020	[d]BV-BRC
Rhodobacter ferrooxidans	WP_008028842	[e]NCBI
Sutterella sp. strain L3_072_000M1_dasL3_072_000M1_concoct_51	JAHALG010000051	[d]BV-BRC
Tortoise microvirus 38	QCS36979	[e]NCBI
Tortoise microvirus 47	QCS37059	[e]NCBI
Tortoise microvirus 70	QCS37215	[e]NCBI
Tortoise microvirus 75	QCS37248	[e]NCBI
Tortoise microvirus 78	QCS37269	[e]NCBI
TPA_as m	DAZ92337	[e]NCBI
TPA_asm	AXH76114	[e]NCBI
TPA_asm	AXH77443	[e]NCBI
TPA_asm	DAZ92296	[e]NCBI
TPA_asm	DAZ92300	[e]NCBI
TPA_asm	DAZ92304	[e]NCBI
TPA_asm	DAZ92308	[e]NCBI
TPA_asm	DAZ92312	[e]NCBI
TPA_asm	DAZ92320	[e]NCBI
TPA_asm	DAZ92324	[e]NCBI
TPA_asm	DAZ92347	[e]NCBI
TPA_asm	DAZ92352	[e]NCBI
Treponema sp. strain Co191P1bin8	WQSF01000016	[d]BV-BRC
Treponema sp. strain Emb289P3bin117	WQWU01000003	[d]BV-BRC
Treponema sp. strain Mgla_MAG_20-bin_15	JAAVAP010000013	[d]BV-BRC
uncultured Escherichia sp. strain MGBC113473	CAJTTP010000085	[d]BV-BRC
Uncultured marine virus clone SOG0069	JX904102	[e]NCBI
uncultured Prevotella sp. strain HGM04906	CAAGFK010000198	[d]BV-BRC
uncultured Prevotella sp. strain HGM05059	CAAFKQ010000288	[d]BV-BRC
uncultured Treponema sp. strain SRR9217393-mag-bin.10	CAJPNP010000015	[d]BV-BRC

[a]François Enault - Université Clermont Auvergne, Clermont-Ferrand, France
[b]Roux et al. *PloS one* **2012**, *7*, e40418, doi:10.1371/journal.pone.0040418.
[c]Metavir - http://metavir-meb.univ-bpclermont.fr
[d]BV-BRC - https://www.bv-brc.org/
[e]NCBI Protein - https://www.ncbi.nlm.nih.gov/protein/

Table S5. Nucleotide (NT) sequences derived from the L segment of viruses of the genus *Orthobunyavirus* and the corresponding codified RDRP amino acid (AA) sequences

Organism	NT Accession code	AA Accession code	Antigenic group
Lukuni virus strain TRVL 10076	KP792672	AKO90162	Anopheles A
Orthobunyavirus tacaiumaense strain BeAn73	MF497778	ASY08211	Anopheles A
Anadyr virus strain LEIV-13395Mg	KU159766	ANB45710	Bunyamwera
Batai virus strain MM2222	JX846597	AFY52605	Bunyamwera
Maguari virus strain BeAr 7272	KX100105	ARI46622	Bunyamwera
Maguari virus strain OBS 6657	KX100117	ARI46638	Bunyamwera
Ngari virus strain Dakar D28542/4e	KC608152	AGM34029	Bunyamwera
Northway virus isolate 0234	MG544835	AVX27622	Bunyamwera
Playas virus strain 75V5938	KX100126	ARI46650	Bunyamwera
Tensaw virus strain TSV-FL06	FJ943509	ACV95628	Bunyamwera
Tlacotalpan virus strain 61D240	KX100120	ARI46642	Bunyamwera
Bwamba virus strain M459	KJ867184	AIN37018	Bwamba
Infirmatus virus	KY569262	ATI21270	California
Inkoo virus	EU789573	ACF10362	California
Lumbo virus strain SAAr 1881	KX817324	APA28997	California
Serra do Navio virus strain BeAr 103645	KX817333	APA28985	California
Snowshoe hare virus	EU203678	ABW87611.2	California
South River virus strain NJO-94F	KX817336	APA28981	California
Tahyna virus isolate Prototype '92' Bardos	HM036213	ADQ08671	California
Trivittatus virus strain Eklund	KR149249	ALI93835	California
Capim virus strain BeAn8582	KU178980	AMR98956	Capim
Orthobunyavirus guajaraense strain BeAn 10615	KP792663	AKO90159	Capim
Alajuela virus strain MARU 11079	KM272186	AIS74638	Gamboa
Calchaqui virus strain AG 83-1347	KM272183	AIS74637	Gamboa
Gamboa virus isolate GAM_130	KT950265	AMD82975	Gamboa
Pueblo Viejo virus isolate 75V-2621	KX900438	ATB53300	Gamboa
Caraparu virus strain FVB0426	KF254784	AGW82145	Group C
Nepuyo virus isolate TRVL 18462	KM280933	AKB96247	Group C
Orthobunyavirus FSL2923	KF254790	AGW82153	Group C
Ananindeua virus strain BEAN109303	KY013484	APM83095	Guama
Catu virus strain BeH 151	KP792660	AKO90158	Guama
Maprik virus isolate MK7532	NC_026281	YP_009117083	Mapputta
Murrumbidgee virus isolate 934	NC_022595	YP_008709776	Mapputta
Kaeng Khoi virus strain PSC-19	KJ867205	AIN37025	Nyando
Mojui dos Campos virus strain BeAn276121	KJ867202	AIN37024	Nyando
Nyando virus strain MP401	KJ867190	AIN37020	Nyando
Wolkberg virus isolate 2562_SA3	NC_034633	YP_009362987	Nyando

Patois virus	MK896491	QLA46901	Patois
Patois virus isolate 63A49	NC_043690	YP_009667042	Patois
Facey's Paddock virus isolate Aus Ch 16129	KF697138	AHY22330	Simbu
Schmallenberg virus isolate F6	KC355457	AGU16235	Simbu
Leanyer virus isolate AusN16701	HM627178	AEA02985	Simbu
Mermet virus isolate AV 782	KF697153	AHY22335	Simbu
Oya virus strain SC0806	JX983194	AGS56984	Simbu
Perdoes virus isolate BeAn789726	KP691624	AJT39461	Simbu
Sathuperi virus	NC_018461	YP_006590073	Simbu
Utinga virus isolate Be An 84785	KF697154	AHY22336	Simbu
Bahig virus strain EgB 90	KP792654	AKO90156	Tete
Matruh virus strain An 1047-61	KP792693	AKO90169	Tete
Oyo virus	HM639780	AEE01391	Tete
Tete virus strain SaAn 3518	KM972719	AJT55735	Tete
Anhembi virus strain SPAr2984	JN572062	AEZ35255	Wyeomyia
Macaua virus strain BeAr306329	JN572068	AEZ35261	Wyeomyia
Sororoca virus strain BeAr32149	JN572071	AEZ35264	Wyeomyia
Taiassui virus strain BeAr671	JN572074	AEZ35267	Wyeomyia

Table S6. Value ranges of intragroup and intergroup comparisons of viral families of the *Orthornavirae* kingdom. For each subset, amino acid sequences (AA) of the RDRP were compared all-against-all using identity percentage, similarity percentage, ML distance, TM-score, and 3Di-character sequence similarity. Sequences of each subset were also compared to the remaining sequences of the whole dataset to determine the intergroup value range. Mean values and their respective standard errors are also displayed.

**Viral family**	**Value range**				
	**AA identity %**	**AA similarity %**	**ML distance**	**TM-score**	**3Di similarity %**
Amalgaviridae					
Intragroup	10.4-71.7	17.0-81.9	0.3988-4.5451	0.293-0.949	26.7-91.7
Intergroup	0.1-20.0	0.3-34.2	2.8784-7.7397	0.154-0.781	1.2-58.5
Botourmiaviridae					
Intragroup	13.0-47.6	21.6-60.7	0.9411-4.1648	0.473-0.869	28.4-77.9
Intergroup	0.3-21.4	0.3-33.6	3.0208-6.9254	0.172-0.703	4.1-53.4
Leviviridae					
Intragroup	49.9-49.9	64.0-64.0	0.8029-0.8029	0.934-0.934	83.6-83.6
Intergroup	1.1-18.5	2.3-33.1	3.2039-6.2764	0.152-0.609	4.0-47.6
Mitoviridae					
Intragroup	16.7-95.5	27.0-98.7	0.0000-3.2695	0.422-0.978	40.7-91.2
Intergroup	0.1-20.6	0.5-33.6	3.2671-7.9910	0.149-0.626	17.8-51.7
Narnaviridae					
Intragroup	10.2-96.8	14.6-98.0	0.0189-3.3618	0.326-0.901	19.3-86.3
Intergroup	0.0-21.4	0.1-33.4	3.0209-7.5811	0.139-0.703	2.8-53.4
Partitiviridae					
Intragroup	12.6-84.4	23.1-91.7	0.1810-4.0758	0.573-0.990	41.3-95.3
Intergroup	0.0-20.0	0.1-34.2	2.8784-7.6867	0.152-0.781	4.9-58.5
Totiviridae					
Intragroup	1.7-95.9	3.8-97.4	0.00941-4.8497	0.137-0.973	2.7-89.3
Intergroup	0.1-19.4	0.3-33.2	3.2949-7.9911	0.139-0.605	1.2-49.5
Mean ± SD	13.5 ± 7.5	22.1 ± 10.1	4.634 ± 1.173	0.504 ±0.130	38.8 ± 10.5

Table S7. Value ranges of intragroup and intergroup comparisons of distinct *Microviridae* subfamilies/groups. Members of each viral group were defined by a maximum likelihood phylogenetic reconstruction using MAFFT and IQ-TREE 2, resulting in *bona fide* subsets. For each subset, amino acid sequences (AA) of the VP1 were compared all-against-all using identity percentage, similarity percentage, ML distance, TM-score, and 3Di-character sequence similarity, to delineate the intragroup value range for each metric. Sequences of each subset were also compared to the remaining sequences of the whole dataset to determine the intergroup value range. Mean values and their respective standard errors are also displayed.

**Viral family**	**Value range**				
	**AA identity %**	**AA similarity %**	**ML distance**	**TM-score**	**3Di similarity %**
Alpavirinae					
Intragroup	14.1-65.4	23.4-76.6	0.5757-6.2533	0.585-0.921	52.0-88.3
Intergroup	2.1-29.3	2.9-43.4	2.6659-8.9999	0.211-0.809	0.1-71.5
Amoyvirinae					
Intragroup	19.0-76.3	29.0-76.3	1e-06-2.2243	0.504-0.941	41.5-87.0
Intergroup	2.8-24.5	5.0-43.4	3.3299-8.9999	0.200-0.759	0.3-71.5
Aravirinae					
Intragroup	36.6-57.0	50.8-66.6	0.5497-1.7486	0.800-0.903	61.2-84.1
Intergroup	3.1-40.8	5.0-59.7	1.2905-8.1870	0.203-0.898	0.1-80.8
Bullavirinae					
Intragroup	46.6-99.5	60.2-100.0	0.0046-1.2428	0.920-0.998	80.5-97.9
Intergroup	2.5-38.6	4.0-56.2	1.9737-8.9999	0.211-0.940	0.3-83.3
CGM					
Intragroup	44.5-70.3	61.6-80.1	0.3717-1.5330	0.833-0.889	85.7-92.1
Intergroup	2.8-37.7	5.0-56.5	1.7786-8.9999	0.195-0.879	0.3-86.5
Gokushovirinae					
Intragroup	32.6-88.8	48.9-93.1	0.1269-2.3583	0.188-0.893	0.3-94.1
Intergroup	1.8-39.2	2.5-55.7	1.5572-8.9999	0.147-0.875	0.1-81.8
Group D					
Intragroup	52.0-52.0	66.1-66.1	0.9622-0.9622	0.889-0.889	82.6-82.6
Intergroup	5.8-41.4	10.1-56.8	1.5269-7.7852	0.208-0.902	0.2-81.8
Liberivirinae					
Intragroup	39.5-95.3	54.3-97.4	0.0063-1.3594	0.667-0.890	64.9-87.2
Intergroup	0.5-24.5	1.1-43.4	3.3299-8.9999	0.182-0.756	0.3-71.5
Occultatumvirinae					
Intragroup	27.7-68.8	42.7-77.7	0.4935-3.0949	0.800-0.943	72.3-90.5
Intergroup	0.6-37.7	1.4-56.6	1.7786-8.9999	0.165-0.879	0.2-86.5
Pequeñovirus					
Intragroup	34.5-56.6	49.8-73.7	0.9208-2.3602	0.950-0.970	84.6-89.0
Intergroup	2.1-38.6	3.2-56.2	1.9737-8.8341	0.224-0.940	0.1-83.3
Parabacteroidetes					
Intragroup	70.0-70.0	80.7-80.7	0.4128-0.4128	0.926-0.926	89.4-89.4

Intergroup	6.0-48.1	11.1-62.6	1.1730-7.8537	0.225-0.910	0.1-83.3
Pichovirinae					
Intragroup	23.7-73.7	37.3-82.8	0.3616-3.0133	0.812-0.947	62.1-91.4
Intergroup	4.8-48.1	7.3-62.6	1.1730-8.9445	0.208-0.910	0.1-83.4
Reekeekeevirinae					
Intragroup	25.2-52.6	39.1-66.1	1,0454-3.4223	0.435-0.647	34.5-75.1
Intergroup	2.9-20.7	3.3-35.7	4.6568-8.9999	0.174-0.546	0.2-59.2
Roodoodoovirinae					
Intragroup	18.9-61.3	32.2-73.6	0.7622-5.1397	0.491-0.812	59.9-80.3
Intergroup	2.9-24.2	3.3-38.9	3.4603-8.9999	0.185-0.777	0.1-70.9
Stokavirinae					
Intragroup	43.0-98.8	57.2-98.8	0.0039-1.4013	0.884-0.949	77.7-94.5
Intergroup	2.6-40.0	5.0-54.2	1.4781-8.6931	0.229-0.891	0.1-81.8
Sukshmavirinae					
Intragroup	28.3-84.2	42.6-86.4	0.1143-2.5059	0.757-0.898	66.5-89.3
Intergroup	0.5-41.5	1.1-56.8	1.4204-8.9999	0.188-0.902	0.1-82.1
Tainavirinae					
Intragroup	36.3-62.2	52.0-74.8	0.6695-2.1794	0.435-0.852	32.4-87.3
Intergroup	1.8-23.7	3.3-40.8	4.4430-8.9999	0.147-0.737	0.3-73.0
Mean ± SD	20.2 ±10.0	31.8 ± 11.9	5.608 ± 2.153	0.656 ± 0.136	58.3 ± 13.8

Table S8. Value ranges of intragroup and intergroup comparisons of distinct antigenic groups of *Orthobunyavirus*. Members of each viral group were defined by a maximum likelihood phylogenetic reconstruction using MAFFT and IQ-TREE 2, resulting in *bona fide* subsets. For each subset, nucleotide sequences of the L segment or amino acid sequences of the corresponding RDRPs were compared all-against-all using different metrics to delineate the intragroup value range for each metric. Sequences of each subset were also compared to the remaining sequences of the whole dataset to determine the intergroup value range. Mean values and their respective standard errors are also displayed.

Viral family	Nucleotide sequences		Amino acid sequences		
	Identity %	ML distance	Identity %	Similarity %	ML distance
Anopheles A					
Intragroup	60.4-60.4	1.6443-1.6443	57.9-57.9	76.1-76.1	0.9067-0.9067
Intergroup	54.3-59.6	1.9656-2.9685	45.1-53.3	64.6-71.8	1.1666-1.8152
Bunyamwera					
Intragroup	72.9-95.4	0.0494-0.6277	81.4-99.2	91.5-99.9	0.008-0.2462
Intergroup	55.0-66.9	1.0406-2.8082	47.7-67.9	66.0-83.6	0.5722-1.5886
California					
Intragroup	71.6-82.5	0.2606-0.6407	80.5-94.9	91.9-98.9	0.0536-0.2608
Intergroup	55.4-66.2	1.1671-2.5060	48.8-65.2	68.1-81.3	0.6799-1.4943
Capim					
Intragroup	76.3-76.3	0.4530-0.4530	87.4-87.4	95.3-95.3	0.1490-0.1490
Intergroup	55.6-72.4	0.6222-2.7467	45.1-79.8	65.7-90.5	0.2762-1.7713
Gamboa					
Intragroup	77.1-96,9	0.0171-0.3786	92.3-99.6	96.9-100.0	0.0044-0.0741
Intergroup	54.0-59.7	1.7520-2.8972	45.7-55.7	64.6-73.6	1.0962-1.7452
Group C					
Intragroup	71.6-84.3	0.2264-0.6799	76.6-97.9	89.0-99.5	0.0212-0.3387
Intergroup	54.8-68.5	0.9035-2.6671	46.3-70.7	66.0-85.2	0.4782-1.6386
Guama					
Intragroup	75.2-75.2	0.4569-0.4569	88.1-88.1	95.4-95.4	0.1394-0.1394
Intergroup	54.8-72.4	0.6222-2.8799	45.4-79.8	65.0-90.5	0.2762-1.7171
Mapputta					
Intragroup	64.4-64.4	1.2935-1.2935	61.4-61.4	78.9-78.9	0.7883-0.7883
Intergroup	55.2-59.5	1.9962-2.9506	45.4-51.1	65.1-71.2	1.3131-1.8044
Nyando					
Intragroup	60.9-65.9	1.1529-1.6341	57.1-65.0	74.3-81.5	0.6702-1.0126
Intergroup	55.6-62.6	1.4292-2.7131	48.0-59.8	66.2-77.6	0.8647-1.5901
Patois					
Intragroup	93.7-93.7	0.0660-0.0660	99.3-99.3	99.9-99.9	0.0071-0.0071
Intergroup	55.0-67.3	1.0137-2.7896	46.7-67.5	66.1-83.6	0.5754-1.6766
Simbu					
Intragroup	59.0-84.2	0.2158-1.7715	54.7-95.9	73.2-98.7	0.0432-1.0861

Intergroup	54.0-61.4	1.8809-2.9685	45.3-54.1	64.6-72.0	1.1727-1.8152
Tete					
Intragroup	60.8-94.7	0.0574-1.5492	57.5-99.1	75.3-99.5	0.0088-0.9540
Intergroup	54.3-60.0	1.8590-2.8455	45.7-53.6	64.7-71.2	1.2081-1.7607
Wyeomyia					
Intragroup	73.2-77.0	0.4858-0.6608	77.5-85.0	89.6-94.2	0.1869-0.3272
Intergroup	55.8-66.9	1.0406-2.6770	48.3-67.9	66.8-83.6	0.5722-1.6270
Mean ± SD	59.43 ± 5.13	2.066 ± 0.555	53.95 ± 9.41	71.85 ± 6.90	1.249 ± 0.363

Table S9. Data partitioning of a dataset of 55 nucleotide sequences of the large (L) segment of *Orthobunyavirus* viruses using different metrics of the MPACT program.

Metric	Value range	# of clusters	Value range	# of clusters
Nucleotide identity %	95-100	53	70-100	24
	90-100	50	65-100	18
	85-100	50	60-100	9
	80-100	45	55-100	2
	75-100	32	50-100	1
Nucleotide ML distance	0-0.3	45	0-1.8	8
	0-0.6	29	0-2.1	6
	0-0.9	23	0-2.4	1
	0-1.2	16	0-2.7	1
	0-1.5	13	0-3.0	1
Amino acid identity (%)	94-100	45	64-100	17
	88-100	35	58-100	13
	82-100	28	52-100	6
	76-100	24	46-100	1
	70-100	22	40-100	1
Amino acid similarity (%)	96-100	39	76-100	14
	92-100	29	72-100	7
	88-100	25	68-100	1
	84-100	23	64-100	1
	80-100	17	60-100	1
Amino acid ML distance	0-0.2	32	0-1.2	6
	0-0.4	23	0-1.4	2
	0-0.6	20	0-1.6	1
	0-0.8	15	0-1.8	1
	0-1.0	9	0-2.0	1

Table S10. Data partitioning of a dataset of 55 nucleotide sequences of the large (L) segment of *Orthobunyavirus* viruses using the MPACT program with ranges of 65 to 100% of identity and 0 to 1.5 for maximum-likelihood distance as partitioning criteria. To facilitate sequence identification within each partition, sequence codes are color-coded to match the serogroup color scheme used in the corresponding phylogenetic tree (Figure 7).

Nucleotide pairwise identity %		Nucleotide ML distance	
**Cluster**	**Sequence**	**Cluster**	**Sequence**
1	Guama_KP7926601	1	California_KX8173241
	GroupC_KF2547841		California_EU2036782
	GroupC_KF2547901		California_EU7895731
	GroupC_KM2809331		California_HM0362131
	Capim_KP7926631		Bwamba_KJ8671841
	Capim_KU1789801		California_KR1492491
	Guama_KY0134841		California_KX8173331
	Patois_NC_0436901		California_KX8173361
2	Patois_MK8964911		California_KY5692621
3	Tete_KP7926541	2	Gamboa_KT9502651
	Tete_KM9727191		Gamboa_KM2721831
	Tete_KP7926931		Gamboa_KM2721861
4	Tete_HM6397801		Gamboa_KX9004381
5	Simbu_JX9831941	3	Nyando_NC_0346331
	Simbu_KF6971381		Nyando_KJ8672021
	Simbu_KF6971531		Nyando_KJ8672051
	Simbu_KF6971541	4	Nyando_KJ8671901
6	Simbu_KP6916241	5	Bunyamwera_KX1001171
7	Simbu_HM6271781		Bunyamwera_FJ9435091

8	Simbu_KC3554571		Wyeomyia_JN5720621
	Simbu_NC_0184611		Wyeomyia_JN5720681
9	Mapputta_NC_0225951		Wyeomyia_JN5720711
10	Mapputta_NC_0262811		Wyeomyia_JN5720741
11	California_EU7895731		Bunyamwera_JX8465971
	California_EU2036782		Bunyamwera_KC6081521
	California_HM0362131		Bunyamwera_KU1597661
	Bwamba_KJ8671841		Bunyamwera_KX1001051
	California_KR1492491		Bunyamwera_KX1001201
	California_KX8173241		Bunyamwera_KX1001261
	California_KX8173331		Bunyamwera_MG5448351
	California_KX8173361	6	AnophelesA_MF4977781
	California_KY5692621	7	AnophelesA_KP7926721
12	Nyando_KJ8672051	8	Capim_KU1789801
	Nyando_NC_0346331		Group C_KF2547841
13	Nyando_KJ8672021		Group C_KF2547901
14	Nyando_KJ8671901		Group C_KM2809331
15	Gamboa_KM2721861		Guama_KP7926601
	Gamboa_KM2721831		Capim_KP7926631
	Gamboa_KT9502651		Guama_KY0134841
	Gamboa_KX9004381		Patois_MK8964911
16	Bunyamwera_KX1001051		Patois_NC_0436901
	Bunyamwera_FJ9435091	9	Tete_KP7926931
	Wyeomyia_JN5720621		Tete_HM6397801
	Wyeomyia_JN5720681		Tete_KM9727191
	Wyeomyia_JN5720711		Tete_KP7926541
	Wyeomyia_JN5720741	10	Simbu_KF6971531

	Bunyamwera_JX8465971		Simbu_JX9831941
	Bunyamwera_KC6081521		Simbu_KF6971381
	Bunyamwera_KU1597661		Simbu_KF6971541
	Bunyamwera_KX1001171		Simbu_KP6916241
	Bunyamwera_KX1001201	11	Simbu_HM6271781
	Bunyamwera_KX1001261	12	Simbu_NC_0184611
	Bunyamwera_MG5448351		Simbu_KC3554571
17	Anopheles A_KP7926721	13	Mapputta_NC_0262811
18	Anopheles A_MF4977781		Mapputta_NC_0225951

Table S11. Data partitioning of a dataset of 55 amino acid sequences of the RDRP coded by the large (L) segment of *Orthobunyavirus* viruses using the MPACT program with ranges of 64 to 100% of identity percentage, 80 to 100% of similarity percentage and 0 to 0.8 for maximum-likelihood distance as partitioning criteria. To facilitate sequence identification within each partition, sequence codes are color-coded to match the serogroup color scheme used in the corresponding phylogenetic tree (Figure 7).

**Amino acid pairwise identity %**		**Amino acid pairwise similarity %**		**Amino acid ML distance**	
**Cluster**	**Sequence**	**Cluster**	**Sequence**	**Cluster**	**Sequence**
1	California_ADQ08671	1	California_ADQ08671	1	California_APA28981
	California_ABW87611		Bwamba_AIN37018		Bwamba_AIN37018
	California_ACF10362		California_ABW87611		California_ABW87611
	California_ALI93835		California_ACF10362		California_ACF10362
	California_APA28981		California_ALI93835		California_ADQ08671
	California_APA28985		California_APA28981		California_ALI93835
	California_APA28997		California_APA28985		California_APA28985
	California_ATI21270		California_APA28997		California_APA28997
2	Bwamba_AIN37018		California_ATI21270		California_ATI21270
3	Gamboa_AIS74638	2	Nyando_AIN37025	2	Gamboa_AMD82975
	Gamboa_AIS74637		Nyando_YP_009362987		Gamboa_AIS74637
	Gamboa_AMD82975	3	Nyando_AIN37024		Gamboa_AIS74638
	Gamboa_ATB53300	4	Nyando_AIN37020		Gamboa_ATB53300
4	Nyando_AIN37025	5	Gamboa_AIS74638	3	Nyando_YP_009362987
	Nyando_YP_009362987		Gamboa_AIS74637		Nyando_AIN37025
5	Nyando_AIN37024		Gamboa_ATB53300	4	Nyando_AIN37020
6	Nyando_AIN37020	6	Gamboa_AMD82975	5	Nyando_AIN37024

7 Bunyamwera_ARI46622
Bunyamwera_ACV95628
Bunyamwera_AFY52605
Bunyamwera_AGM34029
Bunyamwera_ANB45710
Bunyamwera_ARI46638
Bunyamwera_ARI46642
Bunyamwera_ARI46650
Bunyamwera_AVX27622
Wyeomyia_AEZ35255
Wyeomyia_AEZ35261
Wyeomyia_AEZ35264
Wyeomyia_AEZ35267
8 AnophelesA_AKO90162
9 AnophelesA_ASY08211
10 Capim_AKO90159
Capim_AMR98956
GroupC_AGW82145
GroupC_AGW82153
GroupC_AKB96247
Guama_AKO90158
Guama_APM83095
Patois_QLA46901
Patois_YP_009667042
11 Tete_AKO90156
Tete_AJT55735
Tete_AKO90169

7 Bunyamwera_ARI46622
Bunyamwera_ACV95628
Bunyamwera_AFY52605
Bunyamwera_AGM34029
Bunyamwera_ANB45710
Bunyamwera_ARI46638
Bunyamwera_ARI46642
Bunyamwera_ARI46650
Bunyamwera_AVX27622
Wyeomyia_AEZ35255
Wyeomyia_AEZ35261
Wyeomyia_AEZ35264
Wyeomyia_AEZ35267
8 AnophelesA_ASY08211
9 AnophelesA_AKO90162
10 Mapputta_YP_008709776
11 Mapputta_YP_009117083
12 Capim_AKO90159
Capim_AMR98956
GroupC_AGW82145
GroupC_AGW82153
GroupC_AKB96247
Guama_AKO90158
Guama_APM83095
Patois_QLA46901
Patois_YP_009667042
13 Tete_AKO90156

6 AnophelesA_ASY08211
7 AnophelesA_AKO90162
8 Bunyamwera_ARI46638
Bunyamwera_ACV95628
Bunyamwera_AFY52605
Bunyamwera_AGM34029
Bunyamwera_ANB45710
Bunyamwera_ARI46622
Bunyamwera_ARI46642
Bunyamwera_ARI46650
Bunyamwera_AVX27622
Wyeomyia_AEZ35255
Wyeomyia_AEZ35261
Wyeomyia_AEZ35264
Wyeomyia_AEZ35267
9 Guama_APM83095
Capim_AKO90159
Capim_AMR98956
GroupC_AGW82145
GroupC_AGW82153
GroupC_AKB96247
Guama_AKO90158
Patois_QLA46901
Patois_YP_009667042
10 Tete_AKO90169
Tete_AJT55735
Tete_AKO90156

12	Tete_AEE01391		Tete_AJT55735	11	Tete_AEE01391
13	Simbu_AGS56984		Tete_AKO90169	12	Simbu_AHY22335
	Simbu_AHY22330	14	Tete_AEE01391		Simbu_AGS56984
	Simbu_AHY22335	15	Simbu_AGS56984		Simbu_AHY22330
	Simbu_AHY22336		Simbu_AHY22330		Simbu_AHY22336
	Simbu_AJT39461		Simbu_AHY22335		Simbu_AJT39461
14	Simbu_AEA02985		Simbu_AHY22336	13	Simbu_AEA02985
15	Simbu_AGU16235		Simbu_AJT39461	14	Simbu_YP_006590073
	Simbu_YP_006590073	16	Simbu_AEA02985		Simbu_AGU16235
16	Mapputta_YP_008709776	17	Simbu_AGU16235	15	Mapputta_YP_009117083
17	Mapputta_YP_009117083		Simbu_YP_006590073		Mapputta_YP_008709776
